# Relation of *Helicobacter pylori* Infection and Lifestyle to the Risk of Chronic Atrophic Gastritis: A Cross-Sectional Study in Japan

**DOI:** 10.2188/jea.12.105

**Published:** 2007-11-30

**Authors:** Kazunori Shibata, Masaki Moriyama, Tetsuhito Fukushima, Hiroshi Une, Motonobu Miyazaki, Naohito Yamaguchi

**Affiliations:** 1 Department of Public Health, School of Medicine, Fukuoka University, Fukuoka 814-0180, Japan.; 2 Department of Hygiene and Preventive Medicine, School of Medicine, Fukuoka University, Fukuoka 814-0180, Japan.; 3 Cancer Information and Epidemiology Division, National Cancer Center Research Institute, Tokyo, Japan.

**Keywords:** chronic atrophic gastritis, *Helicobacter pylori*, lifestyle, path analysis, risk factor

## Abstract

To examine the mutual association of risk factors for both *Helicobacter pylori* (*H. pylori*) infection and chronic atrophic gastritis (CAG), a cross-sectional study on 954 residents of a rural town in Japan was conducted. Using an unconditional logistic model, we calculated the odds ratios (ORs) for *H. pylori* infection according to each lifestyle, as well as the ORs for CAG according to each lifestyle and *H. pylori* infection. A significant positive association was observed between *H. pylori* infection and the risk of CAG (OR = 6.29). On the other hand, a significant negative association was observed between high consumption of light-colored vegetables and the risk of CAG (OR = 0.68). We also used a path analysis to examine the direct relations of gender, age, and lifestyle variables to CAG, as well as the indirect relations of these variables to CAG through *H. pylori* infection. Aging had a significantly direct positive association with CAG. Although aging also had an indirect positive association with CAG through *H. pylori* infection, aging had no association with the consumption of light-colored vegetables. The high consumption of light-colored vegetables showed no association with *H. pylori* infection but had a significantly direct negative association with CAG. The results of this study suggest a possibility that high light-colored vegetables consumption contributes to the prevention of CAG.

## INTRODUCTION

Various epidemiological studies have demonstrated a significantly higher risk of gastric cancer in persons with chronic atrophic gastritis (CAG) than in those without CAG^1^^)^. CAG has been recognized as an intermediate stage in the development of gastric cancer^2^^)^. This means that the elucidation and control of risk factors for CAG may help prevent the occurrence of CAG and, consequently, lead to a prevention or reduction in the occurrence of gastric cancer.

Before the discovery of *H. pylori*, lifestyle factors such as smoking, alcohol consumption and dietary habits had been reported to be risk factors for CAG^3^^)^. After the discovery of *H. pylori* in the human stomach in 1983^4^^)^, it was proven to be the most important risk factor for CAG^5^^)^. However, the risk of CAG cannot be explained solely by *H. pylori* infection. Some epidemiological studies have suggested that not only *H. pylori* infection, but also differences in lifestyle variables appear to play an important role as risk factors for CAG^6^^-^^8^^)^.

In Japan, where both the *H. pylori* infection rate and the prevalence of CAG are high compared to other developed countries^9^^-^^11^^)^, it is important to examine the influence of lifestyle underlying these high rates. However, most epidemiological studies in Japan have so far only separately examined risk factors for either *H. pylori* infection or those for CAG^6^^,^^8^^,^^9^^,^^12^^,^^13^^)^ and no studies have yet simultaneously examined the mutual association of risk factors for both *H. pylori* infection and CAG.

In most previous population-based studies, even though the study subjects were originally sampled from different areas^8^^,^^9^^,^^13^^)^, the subjects were evaluated together in order to estimate the risks. These types of studies are useful in understanding the major risk factors at a national level, but they do not help identify the regional risk factors in order to reduce the occurrence of CAG for an individual area. We therefore conducted a cross-sectional study on the general residents of a rural town in Japan to obtain epidemiological evidence to be incorporated in health education programs to prevent *H. pylori* infection and the occurrence of CAG.

## METHODS

### Subjects

The study area was a rural town in a predominantly mountainous region of in Fukuoka Prefecture, Kyushu, Japan. The chief industry is agriculture and the proportion of individuals engaged in agriculture is about six times higher than that of Japan as a whole (40.2% vs.6.5% based on the 1995 census). The standardized mortality ratio (SMR) of gastric cancer in this town is not significantly different from that of Japan as a whole (males 84.8, females 112.9: 1988-1992). The total population of the town was 16,620 in 1993. In this town, annual health screening is conducted for residents aged 30 and over, and a questionnaire survey on lifestyles was administered for these residents from 1988 to 1990^14^^)^. We asked 2,347 residents, who participated at least once in the past in this survey, to cooperate in the present study. As a result, 1,094 residents took the health screening and gave blood samples in 1993. Out of these residents, 21 were excluded because they were over 80 years of age, and 119 were excluded because of insufficient lifestyle data. A final total of 954 residents aged 30-79 years were enrolled as subjects in this study.

### Serological Method

*H. pylori* infection was determined by serum IgG antibodies for *H. pylori* using an enzyme immunoassay (HM-CAP, EPI Inc., USA). Both the sensitivity and specificity of this assay have been reported to be over 80%^15^^,^^16^^)^. The level of serum pepsinogen I (PGI) and pepsinogen II (PGII) in the same samples were measured for identification of CAG using a radioimmunoassay (PG I / PG II RIABEAD, Dainabot Co. Ltd., Tokyo). CAG was determined by both serum PG I<70ng/ml and PG I/ PG II ratio<3.0. The sensitivity and specificity of the criterion have been shown to be 86% and 82%, respectively^17^^)^. Recent epidemiological studies have adopted this criterion, and the reliability has been recognized^6^^,^^8^^)^.

### Statistical Method

Odds ratios (ORs) for *H. pylori* infection according to lifestyle variables were calculated, then, assuming that *H. pylori* infection itself was an additional risk factor, the OR for CAG of each variable was also calculated. These analyses were conducted by using an unconditional logistic model after adjusting for gender and age.

**Figure 1.**
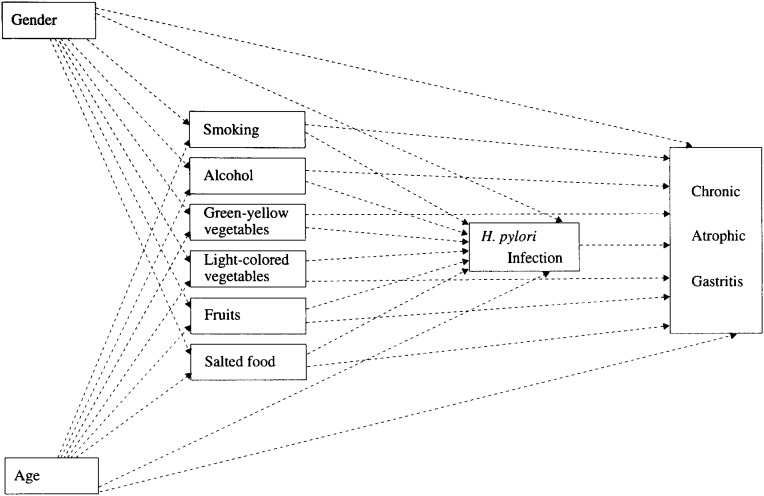
A hypothesized model of gender, age, lifestyle factors, *H. pylori* infection and CAG.

A multivariate analysis using a logistic model can evaluate the influence of each risk factor for *H. pylori* infection or CAG after adjustment for the influence of other factors. However, this method cannot provide a comprehensive evaluation of the mutual relationships among the variables by investigating both the direct influences and the indirect influences. The path analysis can simultaneously evaluate both the direct influence of the independent variable on the dependent variable and the indirect influence of independent variable through other variables, if many relations are set up among the variables. The relationships among the variables are illustrated according to a model prepared in advance (path diagram). This path diagram is prepared according to the epidemiological and medical findings obtained so far. Although the correlations among the variables are actually analyzed, the causal relations are inferred from those correlations according to the model. If more than one pathway is assumed, those pathways are controlled simultaneously, and the existence of causal relations are suggested for pathways between which significant correlations remained. We adopted the path analysis as well taking into consideration these advantages of the path analysis. In the first step, the following relations were defined and analyzed, such as the indirect relations of gender and age to *H. pylori* infection through each lifestyle variable, and the direct relations of gender and age to *H. pylori* infection. *H. pylori* infection is considered to cause CAG. As the second step, we examined the indirect relations of each lifestyle variable to CAG through *H. pylori* infection, as well as the direct relations of gender, age and each lifestyle variable to CAG (Figure 1). The standardized partial regression coefficient obtained by a multiple regression analysis was used as the path coefficient.

In these logistic and path analyses, the following variables, with exception of age (actual value), were assigned as dummy variables: gender (female=0, male=1), *H. pylori* antibody (negative=0, positive=1), presence of CAG (absence=0, presence=1), smoking (non-smoker=0, ex-smoker=1, ≦ 19/day=2, ≧ 20/day=3), alcohol consumption (non-drinker=0, ex-drinker=1, ≦ 3 days /week=2, ≧ 4 days /week=3), and consumption of each food (≦ 3 days /week=0, ≧4 days /week=1). The SAS statistical software package (version 6.11) was used in all statistical analyses.

## RESULTS

Table 1 shows the distribution of the *H. pylori* positive rates and the prevalence of CAG, according to the gender and age group. The *H. pylori* positive rates in males and in females were 80.5% and 70.4%, respectively. The rate in males was significantly higher than that in females (p <0.01). These rates rose significantly as age increased (p <0.01). The prevalence of CAG in males and females was 30.1% and 24.9%, respectively, and no significant difference was found in the rates based on gender. The prevalence also rose significantly as age increased (p <0.01).

**Table 1.** Gender and age distribution according to the positive rates of *H. pylori* antibody and prevalence of chronic atrophic gastritis.

Age group	Number of subjects			Positive rates of *H. pylori* antibody (%)			Prevalence of CAG (%)		
		
	Males	Females	Total	Males	Females	Total	Males	Females	Total
30-39	15	46	61	60.0	47.8	50.8	0.0	6.5	4.9
40-49	40	105	145	62.5	66.7	65.5	12.5	19.1	17.2
50-59	55	182	237	85.5	70.3	73.8	25.5	21.4	22.4
60-69	133	233	366	84.9	73.8	77.9	36.8	27.5	30.9
70-79	69	76	145	82.6	78.9	80.7	37.7	44.7	41.4

Total	312	642	954	80.5	70.4	73.7	30.1	24.9	26.6

				P<0.01	P<0.01	P<0.01	P<0.01	P<0.01	P<0.01

*H. pylori* : *Helicobacter pylori*; CAG: Chronic atrophic gastritis

The test for trend was calculated by a logistic model.

The column on the left in Table 2 shows the OR for *H. pylori* infection according to each lifestyle variable, while the column on the right shows the OR for CAG according to *H. pylori* infection and each lifestyle variable, after adjusting for gender and age. No significant relationship was seen between any of the lifestyle variables and *H. pylori* infection. The OR for CAG in *H. pylori* antibody-positive subjects compared with *H. pylori* antibody-negative subjects was 6.29 (95%CI: 3.73-10.61). The OR for CAG among subjects who consumed light colored-vegetables more than 4 times per week compared with subjects who consumed less than 3 times per week was 0.68 (95% CI:0.52-0.94). No significant relationship was observed between any of the other factors and CAG.

**Table 2.** Gender and age adjusted odds ratio(OR)and 95% confidence interval(95%CI) for *H. pylori* infection and CAG for each factor (n=954).

Factor	n	No. *H.pylori* (+)	OR	(95%CI)	No. CAG (+)	OR	(95%CI)
*H. pylori* antibody							
negative	251	—			17	1.00	
positive	703	—			237	6.29	(3.73-10.61)**

Smoking							
Non-smoker	729	524	1.00		191	1.00	
Ex-smoker	111	90	0.90	(0.46-1.76)	31	0.64	(0.36-1.16)
≦ 19/day	42	36	1.32	(0.51-3.42)	12	0.64	(0.29-1.39)
≧ 20/day	72	53	0.67	(0.33-1.34)	20	0.76	(0.39-1.48)

Alcohol drinking							
Non-drinker	514	364	1.00		135	1.00	
Ex-drinker	24	17	0.80	(0.31-2.05)	7	0.99	(0.38-2.56)
≦ 3days/week	211	158	1.35	(0.92-1.99)	52	1.10	(0.74-1.64)
≧ 4days/week	205	164	1.33	(0.81-2.17)	64	1.11	(0.70-1.77)

Green-yellow vegetables							
≦ 3days/week	363	272	1.00		95	1.00	
≧ 4days/week	591	431	0.95	(0.70-1.29)	159	1.05	(0.78-1.44)

Light colored vegetables							
≦ 3days/week	257	190	1.00		82	1.00	
≧ 4days/week	697	513	1.04	(0.75-1.45)	172	0.68	(0.52-0.94)*

Fruits							
≦ 3days/week	526	393	1.00		136	1.00	
≧ 4days/week	428	310	0.90	(0.67-1.21)	118	1.06	(0.79-1.43)

Salted foods							
≦ 3days/week	801	590	1.00		209	1.00	
≧ 4days/week	153	113	1.02	(0.68-1.52)	45	1.24	(0.84-1.84)

OR : Odds ratio after adjusting for gender and age

*p<0.05, **p<0.01

**Figure 2.**
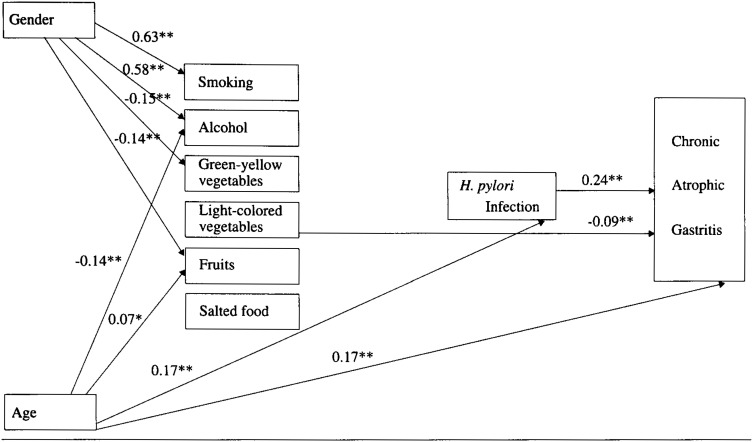
Path estimates of the model with relation to gender, age, lifestyle factors, *H. pylori* infection and CAG(n=954). Continuous arrows (→) represent significant relationships. The degeree of effects are estimated by path coefficients, which are standardized partial regression coefficients. Significant path coefficients are indicated by asterisk (*p<0.05, **p<0.01)

Figure 2 shows the results of the path analysis of a defined multivariate model. The overall F value from the multiple regression analysis of CAG was significant (F=12.91, p<0.001, R^2^=0.1096). The path coefficient between the respective variables was calculated after adjustment for the influence of the other factors.

Being of male gender was found to have not only significantly positive relationships with the high frequencies of smoking and alcohol consumption but also significantly negative relationships with the frequency of consumption of green vegetables and fruits. Aging had a significantly negative relation with the frequency of alcohol consumption and a significantly positive relation with the frequency of fruit consumption. Significantly direct positive relations were observed between aging and *H. pylori* infection and between *H. pylori* infection and CAG. Therefore, aging had an indirect positive relation with CAG through *H. pylori* infection. A significantly direct positive relation between aging and CAG was also observed. Of the six investigated lifestyle variables, none were significantly related to *H. pylori* infection, and only high consumption of light-colored vegetables had a significantly direct negative relation with CAG. Even when the analysis was carried out by limiting the study subjects to those with *H. pylori* infection, a significantly direct negative relation between the consumption of light-colored vegetables and CAG was observed (path coefficient =−0.12, p<0.01).

## DISCUSSION

Regarding the relation between dietary habits and *H. pylori* infection, a few epidemiological studies have suggested a significantly positive relationship between salt intake and the risk of *H. pylori* infection^12^^,^^18^^)^. However, another study showed no relationship between salt intake and an increased risk^7^^,^^19^^)^. As for fruit and/or vegetables consumption, although a few studies showed significant relationships between high consumption of fruit or vegetables^7^^,^^12^^)^, other studies reported non-significant relationships^18^^,^^20^^)^. Therefore, it is difficult to conclude that any specific food contributed to prevention of *H. pylori* as the incompatible findings of previous studies.

While many previous studies reported that no significant relationship was observed between smoking or alcohol consumption and *H. pylori* infection^9^^,^^18^^-^^22^^)^, some previous studies documented significantly positive or negative relations with *H. pylori* infection^12^^,^^13^^,^^23^^-^^25^^)^. In summary, the opinions of investigators concerning the relationships between *H. pylori* infection and smoking or alcohol have not come to agreement yet.

In the present study, both a logistic analysis and a path analysis showed no significant relationship between smoking, alcohol or the consumption of four foods (green-yellow vegetables, light-colored vegetables, fruits, and salted food) and *H. pylori* infection. These results suggest that although these lifestyle variables were not associated with *H. pylori* infection, aging was positively associated with *H. pylori* infection in the study area, which is consistent with many other studies^1^^,^^12^^,^^13^^,^^26^^-^^30^^)^.

Several studies have evaluated the relation of lifestyle variables to the risk of CAG with adjustment for *H. pylori* infection status. The relationship between smoking and CAG is still controversial^6^^,^^8^^,^^31^^-^^33^^)^. The present study does not show any positive association, but rather suggested a slight negative association. This association could be a result of CAG. That is, the manifestation of CAG could cause a person to stop smoking. We found no relation between alcohol consumption and CAG risk, which is highly consistent with other studies^6^^,^^8^^,^^31^^,^^32^^)^.

Regarding green-yellow vegetables consumption, even though one study reported a significantly negative relationship between a high consumption of yellow vegetables and the risk of CAG^8^^)^, other studies showed no relationship between a high consumption of green-yellow vegetables and the risk of CAG^6^^)^. With regard to fruit consumption, while one study suggested a high consumption of fruit decreased the risk of CAG^31^^)^, other studies reported no such relationship^8^^,^^32^^)^. As a result, no definitive conclusions could be made regarding these two foods and the risk for CAG.

In our study, a logistic analysis revealed that a high consumption of light-colored vegetables negatively associated with the risk of CAG. In our path analysis, although aging did have both a direct positive association with CAG and an indirect positive association with CAG through *H. pylori* infection, aging had no relation with the consumption of light-colored vegetables. In addition, whereas a high consumption of this food type had no relation with *H. pylori* infection, a high consumption of this food type had a direct negative association with the risk of CAG. These findings support hypothesis that a high consumption of light-colored vegetables directly suppresses the risk of CAG, independent of either aging or the presence of *H. pylori* infection, both of which are risk factors for CAG.

Comparison of the age-adjusted prevalence of CAG determined on the basis of the same criteria set as in the previous studies in Japan^30^^,^^34^^-^^36^^)^ and the age-adjusted prevalence of CAG in this study showed that CAG prevalence in this area was lower.

The results of analysis in the present study suggested involvement of the light-colored vegetables as a cause of the low CAG prevalence in the investigated area in this study. To verify this suggestion, it is necessary not only to investigate the frequency of consumption of light-colored vegetables per week but also to determine whether consumption of light-colored vegetable is really high in this area by carrying out detailed survey of the amount and kinds of those light-colored vegetables consumed and comparing the results with the findings from the high CAG prevalence areas.

Regarding intake of micro-nutrients, a few studies have shown a high intake of carotene to decrease the risk of CAG^7^^,^^8^^)^. As for vitamin C, several studies have shown a high intake of vitamin C to decrease the risk of CAG^7^^,^^33^^)^.

According to a national nutrition survey in Japan conducted during the same period as this study, rural residents took more vitamin C from light-colored vegetables than from green-yellow vegetables^37^^)^. We therefore consider that light-colored vegetables, which are often eaten raw in this rural area, may suppress the occurrence of CAG through the effect of vitamin C, which is often destroyed during the cooking process, rather than carotene. However, in the present study no significant association was observed for the consumption of either fruit or green-yellow vegetables that also contain vitamin C. Additional research including details on the methods of cooking and/or the intake of micro-nutrients in this area are called for to clarify this discrepancy.

Recently, the hospital based eradication therapy of *H. pylori* has been adopted as a strategy to treat peptic ulcer patients in the Japanese clinical setting^38^^)^, but such preventive eradication therapy for CAG is difficult to implement due to such problems as side-effects, the appearance of *H. pylori* drug resistance, the cost effectiveness and the high infection rate^39^^,^^40^^)^. Therefore, health education regarding dietary habits is considered to be one of the promising methods of community based CAG prevention.

The results of this study suggest a possibility that a high consumption of light-colored vegetables contributes to the prevention of CAG. To clarify this possibility, further epidemiological studies are required in the prospective study design including detailed information of the amount and kinds of the vegetables as well as the methods of cooking the vegetables.
